# The Effect of Cinnamon on Glycolipid Metabolism: A Dose–Response Meta-Analysis of Randomized Controlled Trials

**DOI:** 10.3390/nu15132983

**Published:** 2023-06-30

**Authors:** Tingqing Yu, Kun Lu, Xinyi Cao, Hui Xia, Shaokang Wang, Guiju Sun, Liang Chen, Wang Liao

**Affiliations:** 1 Key Laboratory of Environmental Medicine and Engineering of Ministry of Education, Department of Nutrition and Food Hygiene, School of Public Health, Southeast University, Nanjing 210009, China; ytqing@seu.edu.cn (T.Y.); lukun13023008259@163.com (K.L.); 220214004@seu.edu.cn (X.C.); huixia@seu.edu.cn (H.X.); shaokangwang@seu.edu.cn (S.W.); gjsun@seu.edu.cn (G.S.); 2 Public Service Platform of South China Sea for R&D Marine Biomedicine Resources, The Marine Biomedical Research Institute, Guangdong Medical University, Zhanjiang 524023, China; chenliang094@sina.com

**Keywords:** cinnamon, glycolipid metabolism, randomized controlled trials, dose–response, meta-analysis

## Abstract

(1) Background: The effect of cinnamon on the regulation of glycolipid levels in type 2 diabetic patients is still controversial, and there is a lack of research on the dose–response relationship between cinnamon and glycolipid indicators in type 2 diabetic patients. (2) Methods: This dose–response meta-analysis was performed to explore the effect of the cinnamon intervention on glycolipid metabolism. We conducted a comprehensive database search for literature published before November 2022. Nonlinear models were used for dose–response relationship analysis. (3) Results: We identified that a cinnamon intervention was effective in controlling triglyceride (TG) levels (mean difference = −7.31; 95%CI: −12.37, −2.25, *p* = 0.005) and low-density lipoprotein cholesterol (LDL-C) levels (mean difference = −6.78; 95%CI: −11.35, −2.22, *p* = 0.004) in type 2 diabetic patients; however, it also was able to increase high-density lipoprotein cholesterol (HDL-C) levels in patients with type 2 diabetes (mean difference = 1.53; 95%CI: 1.01, 2.05, *p* < 0.001). However, the cinnamon intervention had no significant effect on the level of fasting blood glucose, glycated hemoglobin (HbA1c), or total cholesterol (TC) levels. We found a significant effect of the cinnamon intervention dose on the TG level (*p*-nonlinearity = 0.016) and LDL-C (*p*-nonlinearity = 0.019) in the nonlinear dose–response analysis. In the subgroup analysis, we found a hypoglycemic effect with the cinnamon dose ≤1200 mg (mean difference = −11.1, 95%CI: −14.64, −7.58, *p* < 0.001). (4) Conclusion: Cinnamon intervention may be beneficial in lowering TG and LDL-C levels while enhancing HDL-C levels, and the dosage of the intervention was an important factor in influencing the TG and LDL-C levels.

## 1. Introduction

Type 2 diabetes is defined as an ongoing decrease in beta-cell insulin production in the context of insulin resistance that develops over time and accounts for 90% to 95% of all diabetes cases [1]. The chronic hyperglycemia of diabetes causes a gradual progression from chronic damage to failure of many vital organs in the body, including serious damage to the eyes, kidneys, heart, and other organs [2]. It was estimated in 2019 that diabetes adversely affects the health status and quality of life of approximately 9.3% of the global population and is anticipated to climb to 10.2% (578 million people) and 10.9% (700 million people) by 2030 and 2045, respectively [3]. Current treatment options for diabetes include non-insulin antidiabetic drug therapy, insulin and insulin analogues therapy, and a combination of insulin and oral hypoglycemic drugs [4]. Although most anti-diabetic drugs show beneficial effects either used alone or in combination, they also have many negative effects; therefore, one of the primary priorities in fighting against this disease is discovering the ideal treatment.

Cinnamon bark is one of the most widely used spices globally, not only in cooking but also as a traditional herbal. Cinnamaldehyde and trans-cinnamaldehyde (Cin) are major important components of cinnamon, both of which are present in cinnamon essential oil [5]. It was found that cinnamaldehyde exerts its anti-hyperglycemic effect by stimulating insulin release, and the mechanisms of action might be similar to that of glibenclamide [6]. Notably, cinnamon-derived water-soluble polyphenol polymers could enhance insulin-dependent in vitro glucose metabolism by approximately 20-fold. Such an effect was thought to be related to the antioxidant activity of the polymers [7].

Diabetes is closely associated with cardiovascular diseases. On the other hand, factors contributing to cardiovascular risks, such as obesity, hypertension, and dyslipidemia, are common in diabetes, and cardiovascular disease is still the primary cause of death in diabetics [8]. Although cinnamon has been reported to have some positive impacts on glucose and lipid metabolism in diabetic individuals, as well as the potential to be employed in the treatment of cardiovascular illnesses, more research is needed to confirm these findings [9]. Especially when used as a therapeutic or complementary medicine [10,11,12], the potential metabolic benefits of cinnamon are still debatable [13]. Collectively, this study systematically reviewed the effects of cinnamon intervention on glucolipid metabolic indexes in type 2 diabetics and added the analysis of dose–response relationships, which sought to provide a more comprehensive understanding of the role of cinnamon in regulating glucolipid metabolism in type 2 diabetics.

## 2. Materials and Methods

This study was planned in conformity with the Reporting Items for Systematic Reviews and Meta-Analysis (PRISMA) 2020 recommended guidance.

### 2.1. Literature Search Strategy

The initial literature search was systematic and comprehensive, all retrieved literature was published before November 2022, and the types of studies covered by the literature were randomized controlled trials. Databases searched included Web of Science, PubMed, the Cochrane Library, and Embase. To conduct the search, terms from titles or abstracts were combined with MeSH. The terms included “Type 2 diabetes OR diabetes mellitus/typeIIOR type 2 diabetes mellitus OR type 2 diabetes OR Diabetes” for the population, and “Cinnamomum zeylanicum OR Cinnamomum verum OR Cinnamon OR Cinnamons” was used for interventions. The references listed in the retrieved publications were also evaluated to locate more relevant studies. Endnote X9 was used for document management.

### 2.2. Inclusion and Exclusion Criteria

Clinical trials that match the criteria given below were included: (1) The study was developed as a randomized clinical trial to look into the effects of cinnamon on blood glucose and lipid metabolism in type 2 diabetic patients; (2) At least one of the following outcome variables should be included in the study: glycosylated hemoglobin (HbA1c), fasting blood glucose (FBG), total cholesterol (TC), high-density lipoprotein cholesterol (HDL-C), low-density lipoprotein cholesterol (LDL-C), and triglycerides (TG); and (3) Insulin was not used during the clinical trial. Any clinical trial that fit one of the following descriptions was excluded: (1) the study looked at the biological benefits of cinnamon in addition to its hypoglycemic and lipid-lowering effects; (2) previous consumption of cinnamon in any form; (3) interventions other than cinnamon were used; and (4) the cinnamon intervention was combined with other food supplements.

### 2.3. Data Extraction

Papers that matched the criteria were eventually included, and two researchers independently assessed and retrieved data from them. The extracted information includes the first author’s last name, publication year, the sample size for each group, country, age range, dosage (mg/d), follow-up period, and type of medication taken. In addition, the standard deviation, and net mean change of fasting blood glucose, glycosylated hemoglobin, total cholesterol, triglycerides, and high-density lipoprotein low-density lipoprotein in the literature were also extracted. For the trial evaluating two different doses of cinnamon, the results were analyzed as two separate trials.

### 2.4. Quality Assessment

The following criteria were used to gauge the quality of the included literature using the Cochrane Collaborative Risk of Bias Tool: (1) creation of random sequences, (2) hiding of allocation, (3) application of blinding to participants and staff, (4) blinding of the result evaluation, (5) inadequate outcome data, (6) selectively reporting outcomes, and (7) additional potential risk biases.

### 2.5. Data Analysis

The International System of Units (IFCC) of glycosylated hemoglobin (mmol/mol) was converted to traditional units (NGSP) (%) using the equation: NGSP = 0.0915 × IFCC + 2.15%. Different units of blood glucose levels and lipid levels were translated to mg/dL (1 mmol/L of TC, LDL-C, and HDL-C was translated to 38.7 mg/dL; 1 mmol/L of TG was translated to 88.5 mg/dL;1 mmol/L FBG was translated to 18 mg/dL). We extracted and organized the data from the literature and then imported them into Stata SE 15.0 software for processing and analysis.

Mean changes from baseline in lipid parameters, blood glucose levels, and glycated hemoglobin levels were considered continuous variables. If changes were not directly reported, calculations were performed using baseline and endpoint data to extrapolate the mean change in outcome from the test.

The threshold for statistical significance was fixed at 0.05. *I*^2^ statistics measured statistical heterogeneity. In general, fixed effects models are used for analysis, when *I*^2^ > 50%, indicating high heterogeneity, the random effects model was adopted. To identify sources of heterogeneity, we analyzed subgroups based on dosage and period of cinnamon intervention.

Sensitivity analysis was utilized to evaluate how specific trials affected the outcome of the study as a whole. To avoid publication bias affecting the authenticity and reliability of the study results, funnel plots were drawn and Egger tests were performed. Wherever the bias existed, the “trim and fill” procedure was used to repair it. In a nonlinear dose–response analysis, fractional polynomial models were used to assess the unique quantitative-effect relationships of cinnamon intervention dose (mg/day) and intervention duration (weeks) in relation to intervention effects [14].

## 3. Results

### 3.1. Literature Selection Process

**Figure 1:**
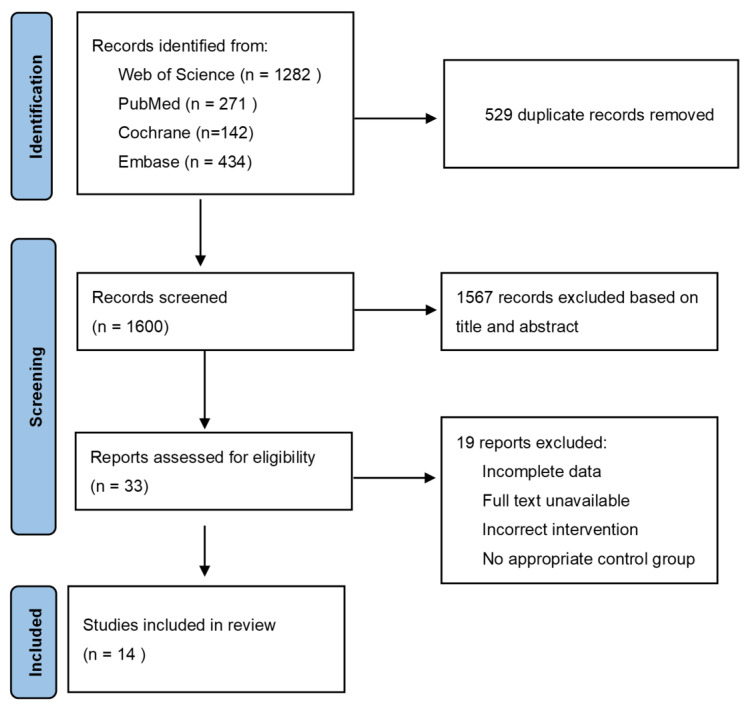
PRISAM flaw chart of selected trails.

After searching four databases, 2129 relevant papers were retrieved, of which, 529 duplicated literatures were excluded. Based on the titles and abstracts, 1567 irrelevant papers were excluded. The qualification review covered a total of 33 pieces of literature, of which, 20 were excluded for the following reasons: (1) incomplete data; (2) full text unavailable; (3) cinnamon was used in combination with other drugs; and (4) placebo was not used in the control group. In the end, a total of 14 pieces of literature with 15 trials were used for the analysis. Figure 1 depicts the complete flow diagram of the selection process.

### 3.2. Study Characteristics

The essential features of the studies examined are summarized in the studies involved in this meta-analysis were published between 2006 and 2021 and involved 965 participants in China, Thailand, the United States, the United Kingdom, Germany, the Netherlands, Israel, and Iran. The mean age of subjects in each trial varied, with the vast majority being in the middle-aged to older age group of 52.1 to 64.4 years. Intervention periods of cinnamon ranged from 6 weeks to 16 weeks, with doses ranging from 120 mg/d to 3000 mg/d (Table 1).

### 3.3. Results of Meta-Analysis

#### 3.3.1. Effects of Cinnamon on the Related Indexes of Glucose Metabolism

**Figure 2:**
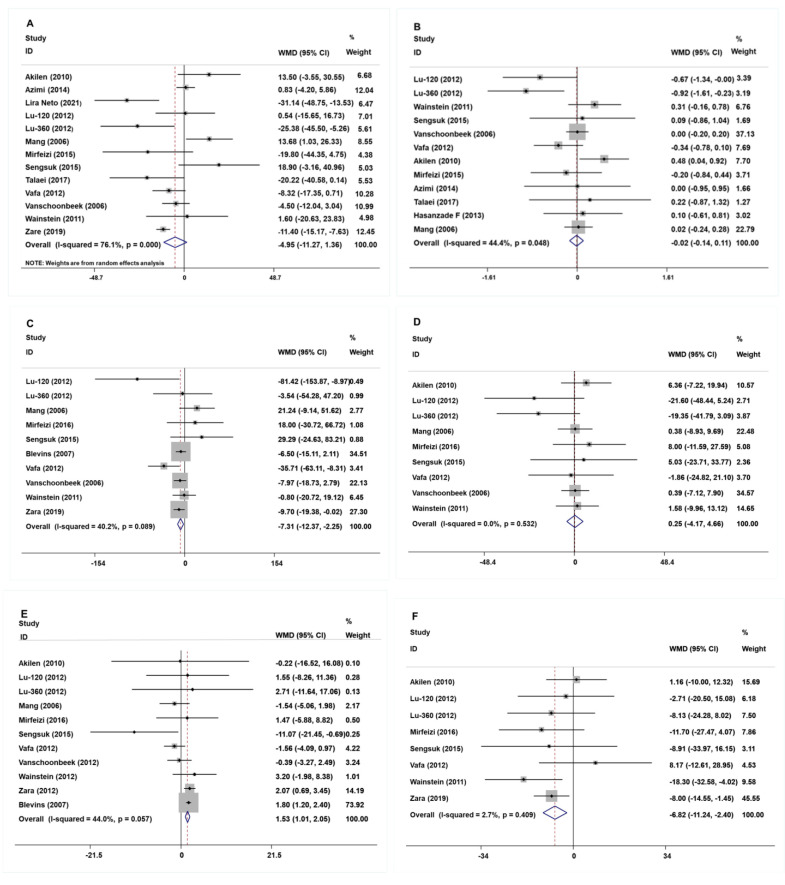
The effects of cinnamon on (**A**) FBG, (**B**) HbA1c, (**C**) TG, (**D**) TC, (**E**) HDL-C, and **(F**) LDL-C. FBG = fasting blood glucose; HbA1c = Glycosylated hemoglobin; TG = Triglyceride; TC = Total cholesterol; HDL-C = High-density lipoprotein cholesterol; LDL-C = Low-density lipoprotein cholesterol [15,16,17,18,19,20,21,22,23,24,25,26,27,28].

**Figure 3:**
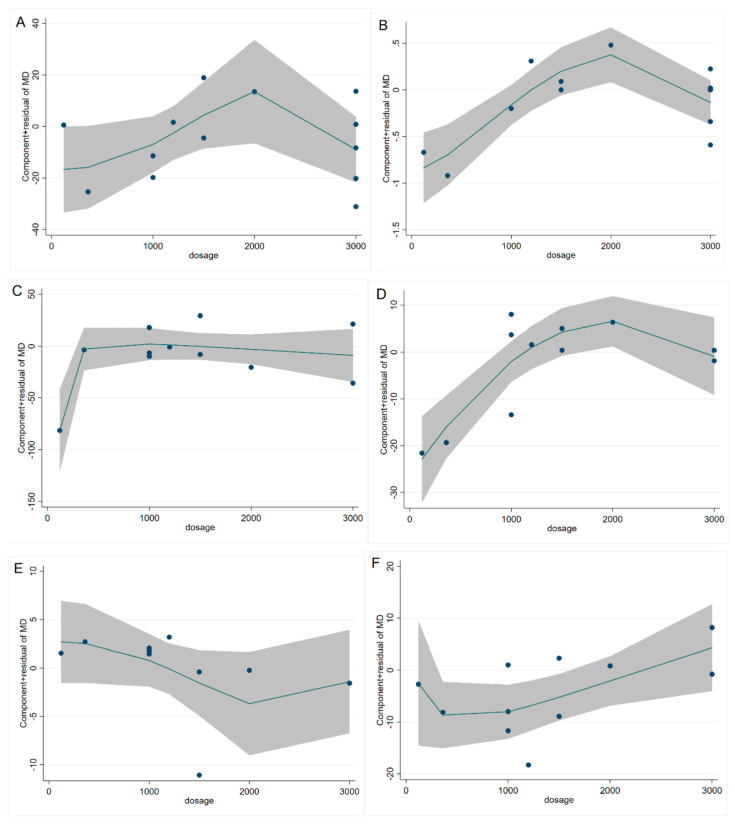
Cinnamon dosage (mg) and unstandardized mean difference (mg/dL) in (**A**) FBG, (**B**) HbA1c, (**C**) TG, (**D**) TC, (**E**) HDL-C, and (**F**) LDL-C. FBG = fasting blood glucose; HbA1c = Glycosylated hemoglobin; TG = Triglyceride; TC = Total cholesterol; HDL-C = High-density lipoprotein cholesterol; LDL-C = Low-density lipoprotein cholesterol.

**Figure 4:**
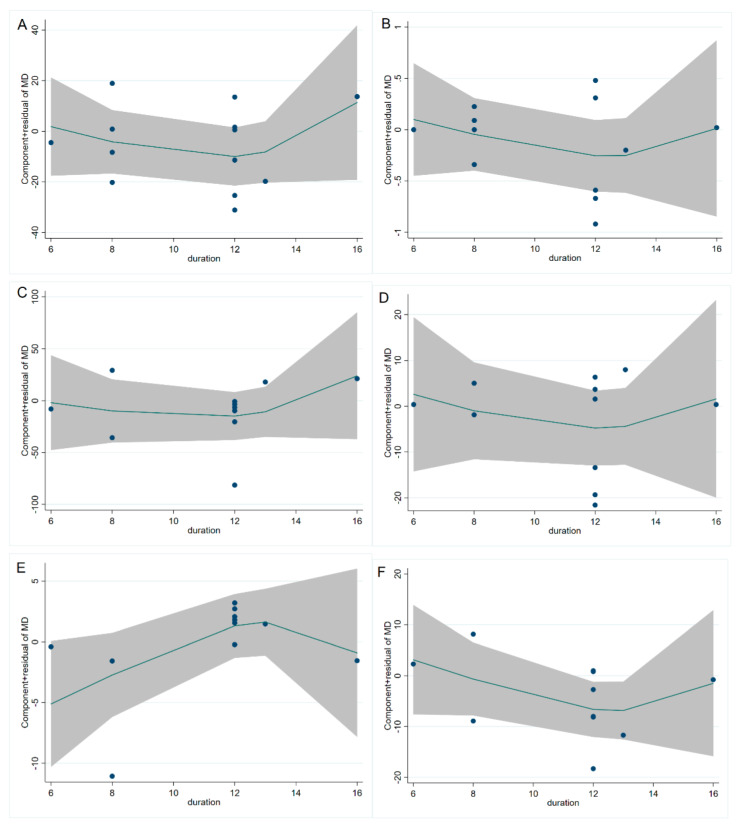
Cinnamon duration (week) and unstandardized mean difference (mg/dL) in (**A**) FBG, (**B**) HbA1c, (**C**) TG, (**D**) TC, (**E**) HDL-C, and (**F**) LDL-C. FBG = fasting blood glucose; HbA1c = Glycosylated hemoglobin; TG = Triglyceride; TC = Total cholesterol; HDL-C = High-density lipoprotein cholesterol; LDL-C = Low-density lipoprotein cholesterol.

The study of fasting blood glucose levels comprised 12 eligible studies with 13 effect sizes. The effect of the cinnamon intervention on FBG levels was not statistically significant (mean difference = −4.95; 95%CI: −11.27, 1.36, *p* = 0.124, *I*^2^ = 76.1%, Figure 2A). Furthermore, the nonlinear dose–response relationship analysis revealed that the dosage (*p*-nonlinearity = 0.180, Figure 3A) and duration (*p*-nonlinearity = 0.420, Figure 4A) of cinnamon supplementation showed no significant effect on the FBG level.

Overall, the level of HbA1c was studied using 11 trials with 12 effect sizes; the results of the fixed effects model revealed that cinnamon intervention had no significant influence on HbA1c level (mean difference = −0.02; 95%CI: −0.14, 0.11, *p* = 0.801, *I*^2^ = 44.4%, Figure 2B). We found that cinnamon intake had no discernible impact on the level of HbA1c in the nonlinear dose–response study (*p*-nonlinearity = 0.159, Figure 3B), and found no discernible effect on the duration of cinnamon intervention (*p*-nonlinearity = 0.616, Figure 4B).

#### 3.3.2. Effects of Cinnamon on Lipid Metabolism-Related Indexes

For the analysis of triglycerides, we included 10 effect sizes from 9 studies, and we found that cinnamon intervention caused a significant reduction in triglyceride levels (mean difference = −7.31; 95%CI: −12.37, −2.25, *p* = 0.005, *I*^2^ = 40.2%, Figure 2C). We noticed a significant non-linear relationship between cinnamon dose and triglycerides levels (*p*-nonlinearity = 0.016, Figure 3C); however, there was no significant non-linear relationship between the supplementation duration and TG level (*p*-nonlinearity = 0.536, Figure 4C).

The effect of cinnamon supplementation on the level of HDL-C was studied in 10 studies with 11 effect sizes, and we found that cinnamon supplementation had significant effects on HDL-C levels (mean difference = 1.53; 95%CI: 1.01, 2.05, *p* < 0.001, *I*^2^ = 44.0%, Figure 2E). Additionally, there was no effect of cinnamon dosage (*p*-nonlinearity = 0.336, Figure 3E) or duration (*p*-nonlinearity = 0.183, Figure 4E) on HDL-C levels.

To summarize the effect of cinnamon on the level of LDL-C, seven randomized controlled trials (eight effect sizes) were pooled and the results indicated a significant effect of cinnamon supplements on LDL-C concentration (mean difference = −6.78; 95%CI: −11.35, −2.22, *p* = 0.004, *I*^2^ = 2.7%, Figure 2F). In addition, we found a non-linear correlation between the dose of cinnamon intervention and LDL-C levels (*p*-nonlinearity = 0.019, Figure 3F); however, the non-linear correlation between duration and LDL-C levels was insignificant (*p*-nonlinearity = 0.527, Figure 4F).

To investigate whether cinnamon interventions improve the level of total cholesterol, eight eligible studies (nine effect sizes) were selected for analysis. The results suggested that cinnamon interventions were not effective in reducing the level of TC level (mean difference = 0.25; 95%CI: −4.17, 4.66, *p* = 0.913, *I*^2^ = 0.0%, Figure 2D). TC based on intervention dosage (*p*-nonlinearity = 0.002, Figure 3D) is affected in a non-linear manner. However, no significant correlation was found for intervention duration (*p*-nonlinearity = 0.788, Figure 4D).

### 3.4. Subgroup Analysis

When the heterogeneity is higher than 50%, the combined results may be unreliable, or the combined results themselves may be inappropriate, at which point we need to determine the source of heterogeneity by subgroup analysis, as indicated in Table 2. With respect to the effect of the cinnamon intervention on FBG, the heterogeneity among studies disappeared when we grouped them according to intervention dosage (*I*^2^ = 29.6%, *p* = 0.224). In these trials, cinnamon intervention ≤1200mg/d significantly reduced FBG levels (mean difference = −11.1; 95%CI: −14.64, −7.58, *p <* 0.001).

### 3.5. Publication Bias and Sensitivity Analysis

**Figure 5:**
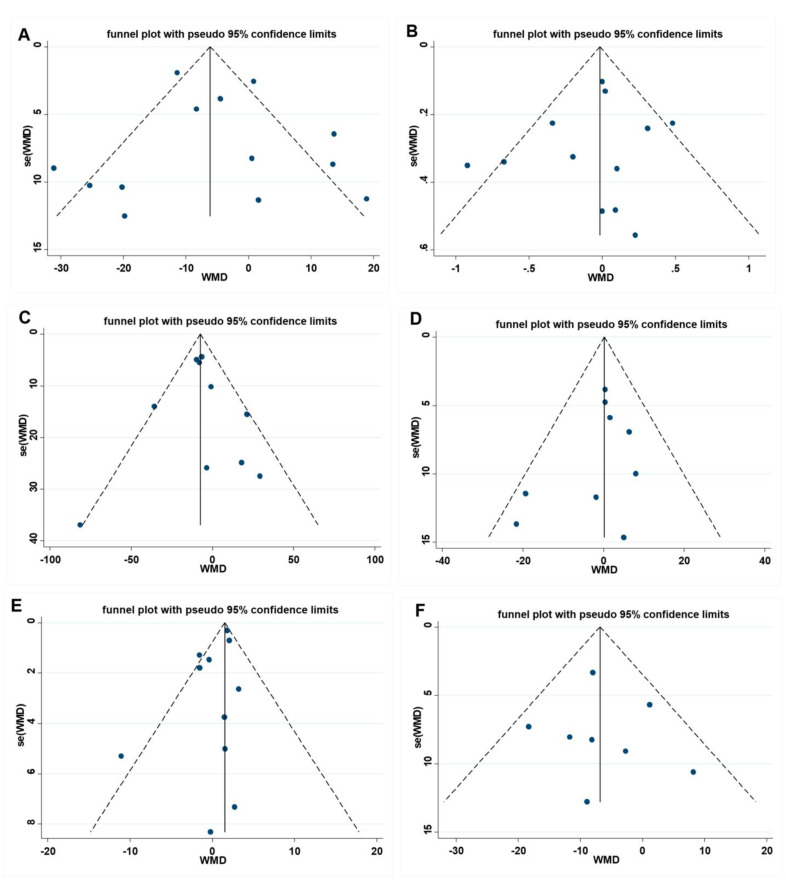
Funnel plot and Egger test for assessing publication bias, (**A**) FBG Egger’s test (*p* = 0.699), (**B**) HbA1c Egger’s test (*p* = 0.576), (**C**) TG Egger’s test (*p* = 0.842), (**D**) TC Egger’s test (*p* = 0.233), (**E**) HDL-C Egger’s test (*p* = 0.103), and (**F**) LDL-C Egger’s test (*p* = 0.092). A = FBG, B = HbA1c, C = TG, D= TC, E = HDL-C, F = LDL-C. FBG= fasting blood glucose; HbA1c = Glycosylated hemoglobin; TG = Triglyceride; TC = Total cholesterol; HDL-C = High-density lipoprotein cholesterol; LDL-C = Low-density lipoprotein cholesterol.

The Egger test and visual funnel plot test were utilized to determine if the research contained any publication bias. As illustrated in Figure 5, the visual funnel plot is roughly symmetrical, suggesting that no publication bias exists, and the Egger test result further confirms that there is no publication bias in FBG (*p* = 0.700), HbA1c (*p* = 0.537), TG (*p* = 0.842), TC (*p* = 0.355), HDL-C (*p* = 0.103), and LDL-C (*p* = 0.759). To ensure that the individual study did not significantly alter the overall study results, we eliminated each paper step-by-step. In this process, no single study was found to have a significant impact on the overall effect.

## 4. Discussion

In this meta-analysis, we discovered that cinnamon intervention contributed to lower triglyceride and LDL-C levels while increasing HDL-C levels. In addition, we noted a decline in fasting blood glucose levels when the cinnamon dose was less than 1200 mg. The cinnamon intervention, however, had no effect on HbA1c or TC. In a non-linear dose–response relationship analysis, we found that the dose of cinnamon intervention had a significant influence on triglyceride and LDL-C levels.

Diabetes is manifested by chronic hyperglycemia caused by insufficient insulin secretion and/or insulin dysfunction. Previous studies have indicated that cinnamon and its extracts can increase insulin sensitivity and ameliorate insulin resistance [29,30]. It has been reported that aqueous extracts of cinnamon could act as dual activators of PPARγ/α and might serve as a substitute for PPARγ agonists in the control of obesity-related diabetes and hyperlipidemia [31]. B-type procyanidin C1 in the cinnamon extract also induces preadipocyte differentiation and functions as a possible enhancer of insulin action in mature adipocytes via the AKT-eNOS pathway [32]. In our study results, the use of cinnamon supplements did not improve the FBG of the subjects, which is different from the results of previous meta-analyses [10,33,34]. However, we found a noticeable drop in the level of FBG at an intervention dose of cinnamon ≤1200 mg in the sub-group analysis. Previous research found a paradoxical effect of the low-dose cinnamon interventions (doses ≤ 1200 mg) on the level of blood glucose, with some studies showing that low-dose cinnamon significantly reduced the level of FBG [20,22,28], while others showed no statistically significant reduction in FBG [27]. Clearly, the results of our analysis support the hypoglycemic effect of low-dose cinnamon, and the exact range of low doses needs to be explored in more well-designed trials in the future.

It has been shown that cinnamaldehyde, the main active ingredient in cinnamon, could improve glycated hemoglobin levels in STZ-induced diabetic rats despite the unclear mechanism [6]. However, cinnamaldehyde is unstable in the human body and may be metabolized to cinnamic acid and converted to cinnamyl alcohol and lose its effect; therefore, the stability of cinnamaldehyde is an important factor affecting its biological activity [35]. We found no significant change in glycosylated hemoglobin levels in the present study. Although some meta-analyses have shown that cinnamon interventions can reduce glycated hemoglobin levels [33,36], which might be due to different inclusion criteria in the literature, such as the inclusion of insulin-using populations, which may confound the actual effects of cinnamon due to the effects of insulin. In addition, the source and form of the cinnamon used can also make a difference in the results.

Type 2 diabetes is associated with a number of interconnected plasma lipid and lipoprotein abnormalities, including low-HDL cholesterol, a predominance of tiny LDL-C particles, and high triglycerides [37]. We found that cinnamon supplementation was effective in lowering LDL cholesterol and raising HDL cholesterol. Previous studies have shown that cinnamic acid could inhibit lipase activity in vitro [38]. The inhibitory effect of lipase activity also restricted the hydrolysis of dietary triglycerides from being absorbed by monoglycerides, whereas free fatty acids were absorbed in the intestine. This effect may help improve the lipid profile of the rats, ultimately resulting in a decrease in LDL-C levels and an increase in HDL-C levels [39].

Cinnamon supplementation was found to be beneficial in decreasing the level of TG in this investigation, but the effect on the total cholesterol level was not found. S-(+)-Linalool is the main component of the leaf essential oil of *C. osmophloeum ct. Linalool*, which significantly reduced blood TG levels and inhibited lipid accumulation by down-regulating 3T3-L1 lipids [40].

In addition, there was a non-linear dose–response association between cinnamon supplementation dosage and changes in TG and LDL-C levels. This suggests that the reduction in TG and LDL-C levels by cinnamon may rely on the dose of the intervention. Interestingly, our analysis yielded results that the cinnamon intervention did not change total cholesterol levels; yet, we observed a non-linear correlation between the dose of cinnamon intervention and the total cholesterol level. We speculate that this phenomenon is caused by the presence of covariance with other factors.

As of now, our study is the first dose–response analysis to explore the effect of the cinnamon intervention on glycolipid levels in type 2 diabetic patients. The findings of our study have a strong causal inference because only RCTs were used in it. In addition, we kept the heterogeneity of the literature included in the study at a low level by sensitivity analysis. However, we could not control the confounding factors among different studies. For example, the species of cinnamon, the form of cinnamon used, the type of medication used by the subjects, dietary habits, and dietary patterns.

According to our meta-analysis, we believe that a daily intake of cinnamon of less than 1200 mg can effectively reduce fasting blood glucose levels, which is an appropriate intervention dose, and the results of related studies also indicate that cinnamon intervention doses of less than 2 g per day or less than 1.5 g per day could exert a more significant effect on blood lipids and blood pressure [41,42]. This result provides new ideas for the design of future clinical trials in this direction, with the aim of finding effective and safe doses of cinnamon for improving glycolipid levels in diabetic patients. Although cinnamon is considered safe by the FDA, adverse reactions, such as gastric upset and rash, as well as itching, have been reported in previous studies [43,44]. Therefore, the dose should be controlled within the safe range when using cinnamon for population interventions.

Although the benefits of cinnamon for diabetic patients are undeniable, and it is inexpensive and has few adverse side effects, cinnamon is not yet sufficient to be a stand-alone treatment for controlling glucose and lipid levels in type 2 diabetic patients, not only because of the conflicting results shown in various clinical trials so far but also due to the unclear mechanisms. In addition, the hypoglycemic and hypolipidemic effects exhibited by cinnamon are not yet well linked to the underlying mechanisms. Future studies may consider exploring the mechanism of action of cinnamon in improving the glycolipid index of patients.

## 5. Conclusions

In conclusion, cinnamon supplementation was shown to improve some glycolipid indicators in diabetic patients. It significantly reduced the levels of TG and LDL-C, but elevated the level of HDL-C and reduced the level of FBG only at doses below 1200 mg in type 2 diabetic patients. In addition, we identified a dose–response relationship between cinnamon intervention and triglycerides and LDL-C, which may provide a reference for future trials.

## Figures and Tables

**Table 1:** Baseline characteristics and interventions of the studies included.

Study	Year	Study Region	Sample Size (I)	Sample Size (C)	Duration	Dose (mg/d)	Age Range (I)	Age Range (C)	Main Characteristics of Medications
Akilen [15]	2010	United Kingdom	30	28	12 weeks	2000	54.90 *±* 10.14	54.43 *±* 12.54	oral antidiabetic
Azimi [16]	2014	Iran	40	39	8 weeks	3000	54.15 *±* 1.0	53.64 *±* 1.3	metformin and glibenclamide
Blevins [17]	2007	United States	29	28	3 months	1000	NA	NA	oral antidiabetic and hypolipidemia
Hasanzade [18]	2013	Iran	35	35	60 days	1000	53.7 *±* 9.7	54.7 *±* 8.1	oral antidiabetic
Lira Neto [19]	2021	United States	71	69	12 weeks	3000	61.7 *±* 11.7	60.8 *±* 10.8	oral antidiabetic
Lu-120 [20]	2012	China	23	20	3 months	120	62.4 *±* 7.9	60 *±* 5.9	gliclazide
Lu-360 [20]	2012	China	23	20	3 months	360	58.9 *±* 6.4	60 *±* 5.9	gliclazide
Mang [21]	2006	Germany	33	32	4 months	3000	62.8 *±* 8.37	63.7 *±* 7.17	oral antidiabetic
Mirfeizi [22]	2016	Iran	27	45	13 weeks	1000	55 *±* 10	54 *±* 12	sulfonylurea, biguanides, and/or thiazolidines
Sengsuk [23]	2015	Thailand	49	50	60 days	1500	57.2 *±* 1.1	56.9 *±* 1.2	oral antidiabetic
Talaei [24]	2017	Iran	20	19	8 weeks	3000	58.9 *±* 7.93	56.26 *±* 9.46	metformin and insulin
Vafa [25]	2012	Iran	19	18	8 weeks	3000	54.11 *±* 10.37	55.67 *±* 7.98	metformin and gliclazide
Vanschoonbeek [26]	2006	Netherlands	12	13	6 weeks	1500	62 *±* 2	64 *±* 2	oral antidiabetic
Wainstein [27]	2011	Israel	29	30	12 weeks	1200	61.7 *±* 6.3	64.4 *±* 15.4	metformin and/or sulfonylurea
Zare [28]	2019	Iran	69	69	12 weeks	1000	52.1 *±* 9.7	53.2 *±* 8.5	oral hypoglycemic agents

NA = Not Available.

**Table 2:** The results of subgroup analysis.

Index	Subgroup	No. of Trials	Mean Difference		*p*	*I*^2^ (%)	*p* Value of Heterogeneity
			Mean	95%CI			
FBG	Cinnamon dose (mg/d)						
	≥1200	8	−2.09	−10.34, 6.16	0.620	76.3	0.001
	≤1200	5	−10.05	−18.07, −2.93	0.007	29.6	0.224
	Duration of the trial (week)						
	≥8	8	−6.56	−17.32, 4.19	0.232	78.2	0.001
	≤8	5	−3.23	−10.31, 3.85	0.371	60.2	0.004

## Data Availability

Not applicable.
